# Relationships between Dream and Previous Wake Emotions Assessed through the Italian Modified Differential Emotions Scale

**DOI:** 10.3390/brainsci10100690

**Published:** 2020-09-29

**Authors:** Francesca Conte, Nicola Cellini, Oreste De Rosa, Antonietta Caputo, Serena Malloggi, Alessia Coppola, Benedetta Albinni, Mariangela Cerasuolo, Fiorenza Giganti, Roberto Marcone, Gianluca Ficca

**Affiliations:** 1 Department of Psychology, University of Campania L. Vanvitelli, Viale Ellittico 31, 81100 Caserta, Italy; orestederosa@outlook.it (O.D.R.); Antonietta.Caputo@unicampania.it (A.C.); alessiacoppola001@gmail.com (A.C.); benedettaalbinni@gmail.com (B.A.); mariangela.cerasuolo@gmail.com (M.C.); roberto.marcone@unicampania.it (R.M.); gianluca.ficca@unicampania.it (G.F.); 2 Department of General Psychology, University of Padova, Via Venezia 8, 35131 Padova, Italy; cellini.nicola@gmail.com; 3 Department of Biomedical Sciences, University of Padova, Via Ugo Bassi 58/B, 35131 Padova, Italy; 4 Padova Neuroscience Center, University of Padova, Via Giuseppe Orus 2, 35131 Padova, Italy; 5 Human Inspired Technology Center, University of Padova, Via Luzzatti 4, 35121 Padova, Italy; 6 Department NEUROFARBA, University of Firenze, Via di San Salvi 12, 50135 Firenze, Italy; serena.malloggi@unifi.it (S.M.); fiorenza.giganti@unifi.it (F.G.)

**Keywords:** dreaming, emotions, positive affect, negative affect

## Abstract

Despite the increasing interest in sleep and dream-related processes of emotion regulation, their reflection into wake and dream emotional experience remains unclear. Here, we aimed to assess dream emotions and their relationships with wake emotions through the modified Differential Emotions Scale (Fredrickson, 2003), which includes a broad array of both positive and negative emotions. The scale has been first validated on 212 healthy Italian participants, in two versions: a WAKE-2wks form, assessing the frequency of 22 emotions over the past 2 weeks, and a WAKE-24hr form, assessing their intensity over the past 24 h. Fifty volunteers from the wider sample completed the WAKE-24hr mDES for several days until a dream was recalled, and dream emotions were self-reported using the same scale. A bifactorial structure was confirmed for both mDES forms, which also showed good validity and reliability. Though Positive and Negative Affect (average intensity of positive and negative items, PA, and NA, respectively) were balanced in dreams, specific negative emotions prevailed; rmANOVA showed a different pattern (prevalence of PA and positive emotions) in wake (both WAKE-2wks and WAKE-24hr), with a decrease of PA and an increase of NA in the dream compared to previous wake. No significant regression model emerged between waking and dream affect, and exploratory analyses revealed a stable proportion of PA and NA (with prevailing PA) over the 3 days preceding the dream. Our findings highlight a discontinuity between wake and dream affect and suggest that positive and negative emotions experienced during wake may undertake distinct sleep-related regulation pathways.

## 1. Introduction

The importance of sleep for healthy emotionality is now widely acknowledged, as testified by the amount of evidence produced on the topic in the last decade (see, e.g., [1,2] for recent reviews). At the physiological level, good sleep appears necessary for the adaptive functionality of the medial prefrontal cortex–amygdala connections, which sustain adaptive emotion regulation processes during wake [3,4,5]. REM sleep, with its peculiar neurophysiology and patterns of neuroanatomical activation, is proposed to play a particularly relevant role in emotional processing. In fact, this sleep state is associated with a relative deactivation of several cortices (inferior parietal, dorsolateral prefrontal and orbitofrontal cortices, posterior cingulate gyrus, and precuneus) [6,7], paralleling an increased activity in subcortical regions (hippocampus, limbic and paralimbic structures, medial prefrontal cortex, basal forebrain, peduncolopontine tegmental nuclei, and anterior cingulate cortex) [6,8]. This pattern of activations, along with the aminergic downtoning occurring during REM sleep [9], and especially the downregulation of noradrenergic input from the locus coeruleus, which has been linked to states of high arousal and stress [10,11], are believed to provide optimal conditions for offline processing of emotional information.

In line with the prominent involvement hypothesized for REM sleep in emotional processing, the most recent theoretical approaches in the field propose an important role of mental activity occurring during sleep (i.e., dreaming, according to Schredl and Wittman’s definition [12]) in these complex regulatory processes. Although the idea of an exclusive link between REM sleep and dreaming has been overcome [13], the preferential relation of dreaming with this sleep state is still put forward in the recent theoretical accounts connecting sleep physiology, sleep mentation, and emotion regulation processes (e.g., [14,15]).

Indeed, several models propose that dreaming actively participates in the regulation of prior wake emotions, by facilitating the resolution of emotional conflicts [16,17], enhancing fear-extinction processes [15], depotentiating the affective tone initially associated with waking events [14]. Another set of hypotheses focus, instead, on the role of dreaming in optimizing affective reactions to future waking events: dreaming would allow an offline simulation of threatening or social episodes and a rehearsal of the corresponding threat- or social coping skills (respectively the “threat simulation theory” [18], and the “social simulation theory” [19]). Ultimately, both types of models converge in suggesting that emotional processing occurring during dreams promotes adaptive behavioral responses to the challenges of waking life.

The idea of a close connection between emotions experienced during wake and those of the dreams is thus central throughout the various models. At the biological level, it is supported by the existence of largely overlapping neural networks sustaining both (REM) dreaming and emotional processing (extensively reviewed in [20]). However, a clear understanding of this relationship and its expression in subjective wake and sleep mentation is still lacking. Indeed, several alternative hypotheses on the presentation of emotions in waking consciousness and dreaming may be put forward. For instance, dream emotions may directly reflect daytime emotions, i.e., the individual predominantly experiences fear and interest during the day, and these appear as predominant during the subsequent dream (whether the dream is recalled or not); another, opposite possibility is that the dream contains emotions that did not receive enough conscious attention during the preceding wake; i.e., fear and interest appear during the dream if, though generated by waking events, they were not sufficiently processed during the day (for numerous possible reasons). Moreover, the possibilities are multiplied when considering emotional valence: in fact, a common mechanism can be hypothesized for the expression in dreams of both positive and negative emotions (e.g., one of the mechanisms proposed above), or else, they could undergo different pathways (e.g., positive emotions dominate in dreams when negative ones are prevalent during wake and vice versa, etc.). Finally, different hypotheses may also be put forward regarding the time span over which these mechanisms unfold: for instance, daytime emotions may be processed in the immediately subsequent dream or with a few days’ lag (in analogy with literature on the “dream lag” and “day-residue” effect [21,22,23]); or each dream may process emotions generated and/or experienced during wider daytime spans (e.g., the last few weeks, the general “time period”), etc.

Some indications may come from the psychological data on dream emotions accumulated up to now. Numerous studies assessed associations between dream emotions (mostly their valence) and several aspects of waking emotionality. For instance, trait anxiety [24], stress [25], and general psychological well-being [26,27] have been found to be related to dream emotional valence, with dream emotional valence corresponding to that of wake. Moreover, higher frequency of negative emotions in dreams has been linked to several psychopathological conditions such as depression [28], anxiety [29], post-traumatic stress disorder [30], and insomnia [31]. Moreover, a few studies have explored the relationships between day-to-day waking life affect and that of subsequent dreams. Some have focused on nightmares or dysphoric dreams, indicating that their frequency is predicted by affect load [32], daily measures of stress [33], and interactions of daily stress and mood with dispositional factors [33,34,35]. In contrast, one study addressing general dream valence (rather than nightmare frequency) did not observe any association between daytime stress level and dream affect [24].

Overall, these results suggest that the predominant valence of waking affect has a tight correspondence with the valence of dream emotionality. However, these data refer to general measures of affective states, which are multicomponential in nature and, though partially overlapping, should be distinguished from the construct of emotion [36]. Specific emotions have been frequently investigated in dream literature, although there are still unsolved discrepancies among the available data, at least partly attributable to the use of self- versus external ratings of dream emotions. In fact, studies using external judgments mostly found fear-related emotions (e.g., fear, anxiety, apprehension) to prevail in dreams [37,38,39]. Instead, while some studies based on subjective ratings found emotions in the fear domain to be the most frequently experienced in dreams (e.g., [40,41]), others showed that joy and approach-related positive emotions (such as interest) may be prevalent (e.g., [42,43,44]). To our knowledge, only three studies [27,45,46] have investigated both daytime and dream specific emotions. Yu [45] studied a set of fifteen emotions in a wide Chinese sample: positive correlations were observed between intensity of pre-sleep, dream, and post-sleep emotions. Gilchrist et al. [27], using an average measure of intensity and duration of each of eight emotions (four positive and four negative), found positive correlations between corresponding wake and dream emotions. Finally, Sikka et al. [46] found that anger and interest ratings were not correlated across wake and dream; also, while their participants experienced more anger in dreams than in the preceding day, interest ratings did not differ between the dream and the previous evening.

However, the paucity of existing data does not yet allow to clearly support any of the aforementioned hypotheses linking waking and dream emotions. Therefore, here we conduct an exploratory study with the aim to investigate the relationships between waking emotions and those of the subsequent night dreams using a self-report instrument, the modified Differential Emotions Scale (mDES [47,48]), which addresses the full array of emotions (both positive and negative). It is worthwhile to note that a main limitation of research on dream emotions may be linked to the use of numerous different instruments for the collection of dream emotion ratings and to the constraints of the most common available scales, such as the POMS (Profile of Mood States [49]), which is limited to maladaptive affective states, or the PANAS (Positive and Negative Affect Scale [50]), which includes positive emotions but exclusively targets high activation affective states. Instead, the mDES, which has seldom been used in dream research [26,43,44,46,51], reduces the risk of underestimating the presence of positive emotions, since it includes a more balanced number of positive and negative items (ten positive and ten negative in the original versions [47,48], twelve positive and ten negative in our Italian version).

In this study, we use three versions of the mDES: in addition to the one assessing dream emotions (DREAM mDES), we adopted two further versions in order to assess wake emotions across two different time frames. The WAKE-24hr form refers to the intensity of emotions in the last 24 h (i.e., the day before the dream) and the other (WAKE-2wks form) refers to the frequency of presentation of each emotion over the past two weeks.

Therefore, the aims of this study are the following:

(a) To investigate the general emotional tone of dreams and the frequency of specific emotions in dreams through a repertoire of emotions broader than the one mostly used in dream literature;
(b) To assess the associations of dream emotions with emotions of the previous day and previous weeks, as well as possible differences in the intensity and frequency of emotions across the dream and the waking periods.

As part of this study, the mDES [47,48] has also been validated on the Italian population.

## 2. Materials and Methods

### 2.1. Participants and Procedure

Four hundred volunteers from the cities of Naples and Caserta (Italy) were screened through a brief ad-hoc interview to collect general demographic data and information on medical condition and life habits. The interview was conducted via telephone by a psychologist of the Sleep Lab of the University of Campania. Two hundred and twelve healthy participants (163 F, 77%; 49 M, 23%; age range: 18–63 years; mean age: 25 ± 8 years) were thus selected for the study, according to the following inclusion criteria: absence of any relevant somatic or psychiatric disorder, no sleep apnea or respiratory disorder symptoms, having a regular sleep-wake pattern, absence of sleep disorders; no history of drug or alcohol abuse, and limited caffeine (no more than 150 mg caffeine per day, corresponding to about three cups of espresso or one cup of American coffee) and alcohol (no more than 250 mL per day) consumption.

The whole selected sample (*n* = 212) participated in the first part of the study, i.e., the validation of the Italian version of the modified Differential Emotions Scale (mDES [47,48]). The two forms of the questionnaire (WAKE-24hr and WAKE-2wks) were developed using standard forward and backward translation procedures [52] and were administered to participants along with the Mannheim Dream Questionnaire (MADRE [53]), to collect data on dream recall frequency and several variables related to dreams, and the Pittsburgh Sleep Quality Index (PSQI [54]) to assess habitual subjective sleep quality.

Of the 212 participants included in the validation study, 50 (38 F, 76%; 12 M, 24%; age range: 19–52 years; mean age: 24.6 ± 6.4 years) volunteered to take part in the second part of the study, i.e., the assessment of associations between waking and dream emotions. Participants received ten copies of the mDES WAKE-24hr version, with the instruction to fill one in each night at bedtime, referring to the emotions experienced during that particular day. This had to be done until the day they recalled a dream. On the morning they recalled a dream, they had to fill in the DREAM mDES, specifically referring to the emotions experienced during the dream. Data collection was thus ended as soon as the mDES ratings of one dream were provided by each participant.

The study design was submitted to the Ethical Committee of the Department of Psychology, University of Campania “L. Vanvitelli”, which approved the research (code 1/2017) and certified that the involvement of human participants was performed according to acceptable standards.

### 2.2. Instruments

(a) Italian version of the modified Differential Emotions Scale (see Appendix A): The original modified Differential Emotions Scale (mDES [47,48]) consists of 20 items corresponding to 20 different emotion categories (10 positive and 10 negative) whose intensity over the past 24 h is rated on a five-point Likert scale (from 0 = Not at all, to 4 = Extremely). Each category is described by three adjectives (e.g., “Grateful, appreciative or thankful”): for clarity purposes, throughout the manuscript, the noun referring to the first of the three adjectives will be used to identify specific emotion categories (e.g., “Gratefulness”). The scale has been validated on the Greek population [55] and has shown to have good psychometric properties in its various translations [26,56,57]. Fredrickson’s most recent version of the questionnaire [48] has been here translated into Italian and supplemented with two additional positive emotions (“sexual/desiring/flirtatious” and “sympathy/concern/compassion”), which were included in the earlier version of the instrument [47]. This standard version is here labeled WAKE-24hr mDES, in order to distinguish it from the other version of the mDES (WAKE-2wks form), assessing the frequency of each emotion over the past two weeks, which was also developed as part of this study following Fredrickson et al.’s suggestion [48]. Furthermore, a specific version has been created (DREAM mDES) for the evaluation of the intensity of dream emotions: it consists of a WAKE-24hr version of the mDES in which instructions are slightly modified, requiring the participant to refer to the emotions experienced during the last recalled dream rather than the past 24 h. The specific instructions provided in the DREAM and the WAKE-24hr mDES versions are the following: “Please think back to how you have felt during your last recalled dream/last 24 h. Using the 0–4 scale below, indicate the greatest amount that you’ve experienced each of the following feelings.” As for the WAKE-2wks form, the instructions are the following: “Please think back to how you have felt during the past two weeks. Using the 0–4 scale below, indicate the frequency with which you’ve experienced each of the following feelings.” (from 0 = Never, to 4 = Very frequently). Finally, the mDES also allows the use of aggregate measures of positive and negative emotionality (the Positive Affect (PA) and Negative Affect (NA) subscales, i.e., average scores of the positive and negative emotion items, respectively), which have shown to have high internal reliability, ranging from 0.82 to 0.94 [58,59].
(b) Pittsburgh Sleep Quality Index (PSQI [54]): This questionnaire assesses sleep quality and disturbances over a 1-month time interval. It yields a global score, ranging from 0 to 21, with higher scores indicating worse sleep quality.
(c) Mannheim Dream Questionnaire (MADRE [53]): This questionnaire measures several variables related to dreams such as frequency of dream recall, nightmares and lucid dreaming, attitude towards dreams and the effects of dreams on waking life. Here we only report data on dream recall frequency, emotional intensity of dreams, affective tone of dreams, and nightmare frequency.

### 2.3. Data Analysis

As for the validation study, the structure and psychometric properties of the Italian version of the mDES (both WAKE-24hr and WAKE-2wks versions) were examined through an item analysis (mean, standard deviation, skewness, and kurtosis), a Principal Components Analysis (PCA) with Varimax Rotation, a confirmatory factor analysis, and a validity and reliability Analysis (Cronbach alpha and inter-item correlations). Descriptive and inferential statistics were analyzed through SPSS Statistics 19 and STATISTICA 10; validity and reliability analyses, as well as PCA, were performed by means of STATISTICA 10; confirmatory factor analysis was carried out through LISREL 8.71.

For the dream study, data analysis was conducted using JAMOVI 1.2.27. Descriptive data are reported as mean ± standard deviation or frequency. To assess the differences in positive and negative emotionality (Positive and Negative Affect subscales) experienced during wake and dream, we conducted a 2 (Valence: Positive/Negative) × 3 (Condition: WAKE-2wks/WAKE-24hr/Dream) repeated measures ANOVA, reporting η_p_^2^ as a measure of effect size and using the Holm test for post-hoc analysis. A series of repeated measures ANOVAs (with 3 levels: WAKE-2wks/WAKE-24hr/Dream) was also performed to explore changes between wake and dream in all the specific emotions, using η_p_^2^ as a measure of effect size and the Holm test (which corrects *p*-values for multiple comparisons) for post-hoc analysis. Moreover, in order to explore the potential predictors of dream emotions, we conducted several multiple linear regressions with each dream emotion as dependent variable and WAKE-24hr and WAKE-2wks emotions as predictors. For each significant predictor, we reported the unstandardized (b) and the standardized (β) coefficients. For both the ANOVAs and the regression models involving specific emotions, main effects were corrected using the False Discovery Rate (FDR) approach with Benjamini–Hochberg adjusted *p*-value, whereas post-hoc tests were corrected using the Holm approach.

Even though more than one WAKE-24hr mDES per participant was collected (due to the lag with which participants remembered a dream since the beginning of data collection), these analyses included only the scale referring to the 24 h preceding the dream (a single WAKE-24hr mDES per participant). Instead, the additional WAKE-24hr mDES scales (referring to 2 and 3 days before the dream, collected only for 20 participants) were studied in a separate analysis aimed to explore the presence of potential lag effects in the expression of waking emotions in the dream. Taking into account the different number of observations collected for each participant, we built a linear mixed model on emotion intensity scores with Valence (Positive and Negative) and Lag (WAKE-24hr-3 Days, WAKE-24hr-2 Days, WAKE-24hr-1 Days, Dream) as fixed factors and participant as random factor. In this case, Bonferroni was used for post-hoc comparisons. Again, *p*-values were corrected using Benjamini–Hochberg adjusted *p*-value, whereas post-hoc tests were corrected using the Holm approach.

A *p* < 0.05 was considered statistically significant.

## 3. Results

### 3.1. Validation of the Instrument

A bifactorial structure was confirmed for both mDES forms, which also showed good validity and reliability. All the results from the validation study are presented as Supplementary Materials.

### 3.2. Descriptive Data of the Dream Study

Average PSQI scores (*n* = 50) were 5.98 ± 2.59, indicating a mild degree of poor subjective sleep quality in our sample.

Data from the MADRE questionnaire, also referring to the 50 participants of the dream study, yielded the following results on dream recall frequency: 5 participants reported to dream almost every day, 15 several times a week, 12 at least once per week, 8 two or three times a month, 5 at least once a month, 4 less than once a month, and 1 never. Emotional intensity of dreams (from 0 = not at all to 5 = very intense) had a median of 2.50 (range 1–4), whereas affective tone had a median of 0 (from −2 = very negative to 2 = very positive), with 38% of participants reporting negatively toned dreams and 48% positively toned dreams. Finally, 58% of the sample reported to experience nightmares at least once a month, whereas the remaining 42% less than once a month.

A total of 50 dream mDES and 50 WAKE-2wks mDES (one per participant) were collected. As for the WAKE-24hr version, 84 scales were collected in total (50 referring to the day immediately preceding the dream and the remaining referred to the previous days); in fact, thirty participants (60%) recalled a dream after 1 night, 6 participants (12%) after 2 nights, and the remaining 14 (28%) after 3 nights.

### 3.3. Characteristics of Dream Emotions

Scores at the Positive and Negative Affect subscales (PA and NA, respectively) of the DREAM mDES did not differ (PA: 0.96 ± 0.74 vs. NA: 1.25 ± 0.80; t98 = −156, *p* = 0.125, Cohens’ d = 0.22), indicating a balanced intensity of positive and negative emotionality in the dream.

**Figure 1:**
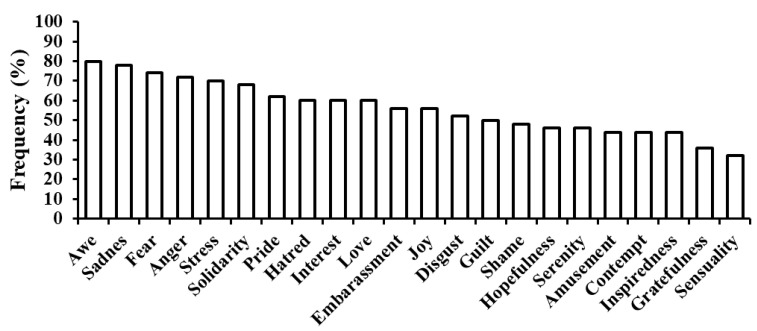
Proportion of participants reporting each of the 22 emotions during the dream.

Looking at the specific emotions, all dreams contain at least five emotions and all of the 22 emotions are reported at least once. On average, participants reported 12.38 ± 4.53 dream emotions. As displayed in Figure 1, the most frequent emotion is Awe (reported by 80% of the participants), followed by Sadness (78%) and Fear (74%), while the least frequent is Sensuality (32%), followed by Gratefulness (36%).

**Figure 2:**
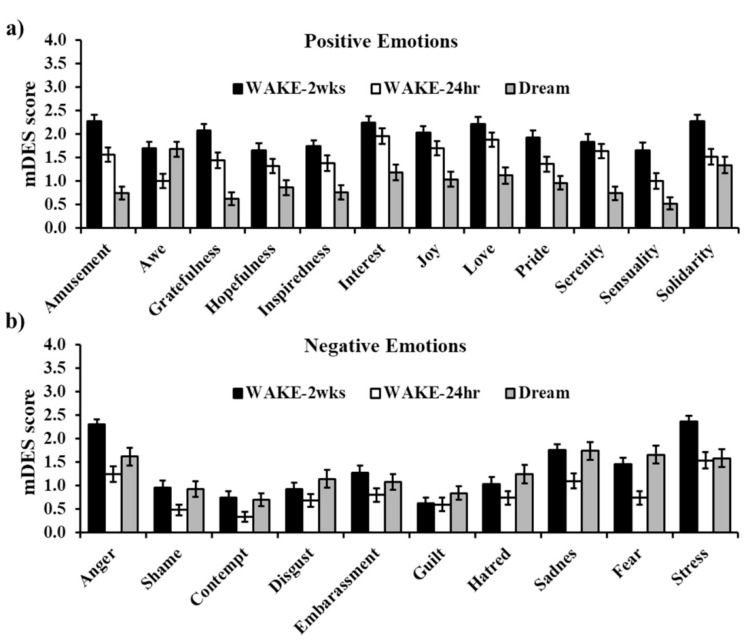
Scores of the 22 emotions in the WAKE-2wks, WAKE-24hr, and Dream mDES. (**a**) and (**b**) display positive and negative emotions, respectively. Error bars represent standard error of the means.

Although PA and NA scores did not differ, the most intensely experienced emotions during the dream were mostly negative (Figure 2b): Sadness (1.74) was followed by Awe (1.68 ± 1.11), Fear (1.66 ± 1.36), Anger (1.62 ± 1.33), and Stress (1.58 ± 1.33).

### 3.4. Differences between WAKE-2wks, WAKE-24hr, and Dream Emotions

**Figure 3:**
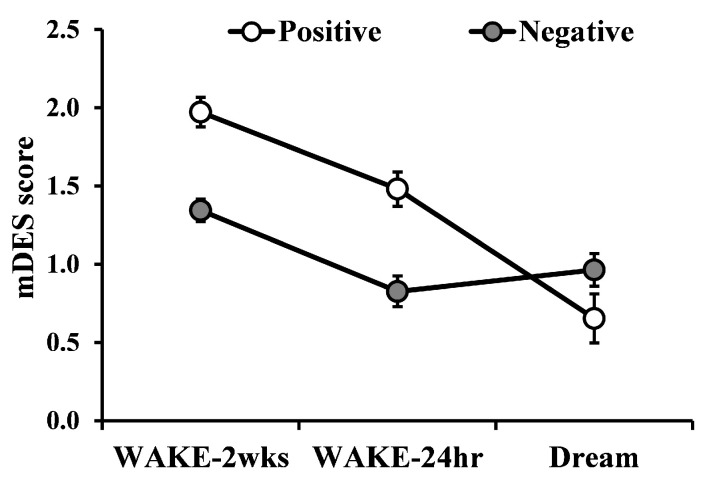
Change in mDES scores as a function of Condition (WAKE-2wks, WAKE-24hr, and Dream) and Valence (Positive and Negative). Error bars represent standard error of the means.

The ANOVA showed a significant main effect of Condition (F_2,98_ = 49.24; *p* = 0.008; η_p_^2^ = 0.50), with higher scores for WAKE-2wks emotions compared to WAKE-24hr and Dream (all *p’s* < 0.001) but no difference between WAKE-24hr and Dream (*p* = 0.467). We also observed a significant main effect of Valence (F_1,49_ = 5.94; *p* = 0.030; η_p_^2^ = 0.11), with higher scores for PA (*p* = 0.003), and a significant interaction Valence × Condition (F_2,98_ = 24.63; *p* = 0.004; η_p_^2^ = 0.33; see Figure 3), with a linear decrease in PA scores from WAKE-2wks to WAKE-24hr and Dream (all *p’s* < 0.001), whereas NA was reduced from WAKE-2wks to WAKE-24hr (*p* < 0.001) and increased from WAKE-24hr to Dream (*p* = 0.004). See also Table 1 for mean and SD of scores at the PA and NA subscales as well as Holm post hoc comparisons between PA and NA scores in the three mDES forms.

Results on specific emotions paralleled those on PA and NA subscales (see Table 2 and Figure 2). All positive emotion scores showed a significant decrease from WAKE-2wks and WAKE-24hr to Dream except Awe and Solidarity. In addition, most positive emotions also significantly decreased from WAKE-2wks to WAKE-24hr, except Interest, Serenity, Hopefulness, Joy and Love (Figure 2a). Negative emotions displayed a more complex pattern. In general, in line with positive emotions, negative emotion scores were lower in the WAKE-24hr compared to the WAKE-2wks mDES (Figure 2b), although this difference was non-significant for Disgust, Guilt, Hatred, and Sadness (Table 2). However, at variance with positive emotions, negative emotions showed higher intensity in the Dream compared to the previous day (WAKE-24hr mDES), often reaching, in the Dream, similar scores to those of the WAKE-2wks scale (Figure 2b), though some of these differences were non-significant (Table 2). Accordingly, most negative emotions did not show differences between WAKE-2wks and Dream scores.

### 3.5. Predictors of Dream Emotions

The linear regression on the PA subscale showed that neither WAKE-2wks nor WAKE-24hr scores were predictive of PA experienced during the dream (F_2,47_ = 1.38, *p =* 0.262, Adj. *R*^2^ = 0.06).

The regression on NA scores was also non-significant (F_2,47_ = 1.89, *p =* 0.161, Adj. *R*^2^ = 0.07).

As for specific dream emotions, the linear regression was significant for Embarrassment (F_2,47_ = 3.42, *p =* 0.048, Adj. *R*^2^ = 0.09) and Interest (F_2,47_ = 4.97, *p =* 0.032, Adj. *R*^2^ = 0.11), with the corresponding WAKE-2wks emotions being the only significant predictors (Embarrassment: *b* = 0.35, 95%CI = 0.04–0.76; *β* = 0.35, *p* = 0.037; Interest: *b* = 0.41, 95%CI = 0.06–0.75; *β* = 0.36, *p* = 0.032). Moreover, significant regression models emerged for Hopefulness (F_2,47_ = 3.77, *p =* 0.037, Adj. *R*^2^ = 0.10), with the WAKE-24hr corresponding emotion as the only significant predictor (*b* = 0.48, 95%CI = 0.13–0.84; *β* = 0.47, *p* = 0.015) and for Pride (F_2,47_ = 3.74, *p* = 0.032, Adj. *R*^2^ = 0.11), but no significant predictor emerged.

### 3.6. Lag Effects

**Figure 4:**
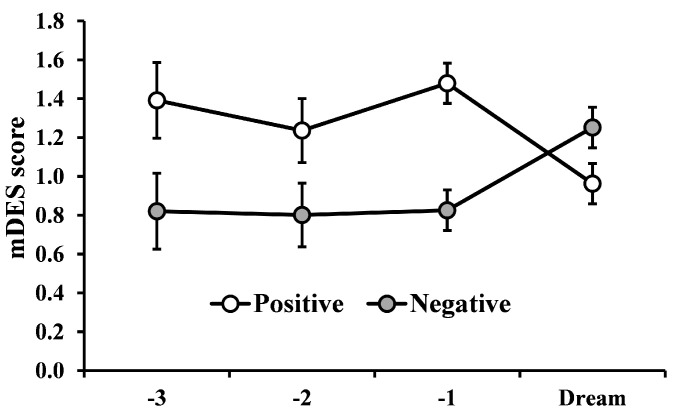
Change in emotion intensity as a function of the lag (WAKE-24hr-3 Days, WAKE-24hr-2 Days, WAKE-24hr-1 Days, Dream) and Valence (Positive and Negative). Error bars represents standard error of the means.

The linear mixed model showed a significant main effect of Valence (F_1,214_ = 11.67, *p <* 0.003), with a general higher intensity for PA, no main effect of Lag (F_3,239_ = 0.32, *p =* 0.806) and a significant interaction Valence × Lag (F_3,214_ = 8.28, *p <* 0.002, Figure 4), indicating a stability of positive and negative emotionality during the 3 days preceding the dream and confirming the decrease of PA and increase of NA in the dream, already observed through our previous rmANOVA.

## 4. Discussion

This study investigated the relationships between dream emotions and those experienced during the previous waking days (both in the day before the recalled dream and over the two weeks preceding it). Emotions were measured through a self-report instrument, the modified Differential Emotions Scale (mDES [47,48]), which addresses a broad array of emotions (both positive and negative). As part of our study, the mDES has also been translated into Italian and validated on the Italian population in two versions: the WAKE-24hr form, referring to the past 24 h, and the WAKE-2wks form, referring to the past two weeks.

### 4.1. Psychometric Properties of the mDES and General Observations on the Instrument

The validation study showed that the Italian mDES, both in its WAKE-24hr and WAKE-2wks forms, has good psychometric properties, in terms of internal validity, construct validity, and reliability. The factorial structure of the instrument was analyzed through confirmatory factor analysis which confirmed the bidimensionality of the scale, with the two factors corresponding to positive and negative emotion categories. The use of the Positive and Negative Affect subscales (already proven to have good internal consistency in previous research [58,59]) thus appears justified also in an Italian sample. The availability of these two subscales, as composite but synthetic measures of general emotional valence, in addition to measures of a broad repertoire of specific emotions, renders the mDES particularly flexible and adaptable to different research aims and approaches.

Moreover, here we show that the mDES may be a useful and reliable tool to investigate dream emotionality. The mDES, in fact, has been very seldom used in dream research-with Sikka et al.’s work [26,43,44,46,51] being, to our knowledge, the only case, whereas most of the studies exploited instruments addressing narrower sets of emotions or ad hoc scales lacking validation (e.g., [27,41,42,45]). We believe that the use of a broader array of emotion items appears particularly appropriate in investigating dream emotionality. Indeed, dream emotions are seldom spontaneously mentioned and/or described in dream reports; as a matter of fact, studies using external judges (who necessarily rely only on explicitly mentioned emotions) typically underestimate the presence of emotions compared to those based on self-ratings (e.g., [43,44,60]). This observation suggests that dream events, characters, and contexts are more salient to the dreamer (and thus spontaneously and explicitly recalled) than the accompanying feelings (although note that this type of narration, centered on what happened, when, where, and who were the actors rather than on subjective thoughts and feelings, may not be specific to dreams but rather characterize any description of everyday waking experiences; see [61,62]). Thus, providing the dreamer with the broadest possible array of emotion categories, from which to recognize (rather than freely recall) those actually experienced, appears to be the best experimental strategy when aiming to obtain a detailed description of dream emotions. This line of reasoning applies all the more to the evaluation of the predominant emotional valence of dreams. Indeed, a relevant advantage of the mDES, compared to other more common emotion scales (e.g., the Profile of Mood States [49]), is its inclusion of a wider array of positive emotions, which limits the risk of underestimating them.

It must be acknowledged here that, according to some authors [44,63,64], self-ratings of dream emotions based on emotion rating scales may be biased by demand characteristics of the rating task (i.e., individuals may be primed by answer options) or phenomena such as the positivity offset (i.e., the tendency to experience mildly positive mood most of the time [65]); still, several authors argue that self-ratings more validly represent dream emotional experiences [39,66,67]; see also [44] for an extensive discussion of pros and cons of self- and external dream emotion ratings). In any case, as shown by Sikka et al. [43,44,51], the mDES may also be usefully employed by external judges as a wide checklist of emotional categories to classify emotions in dream reports. Again, the wide repertoire of positive emotions included in the mDES can help limit the difficulties linked to the more diffuse nature of positive emotions [68], which may make them more difficult to correctly identify and differentiate even by external coders.

### 4.2. Frequency and Valence of Dream Emotions

The number of emotions reported by our participants is in line with several previous studies based on self-ratings of dream emotions, suggesting that most dreams are emotional, i.e., contain at least one emotion: 90.6% of laboratory dreams and 98.4% of home dreams in St-Onge et al. [69] and 100% in Sikka et al. [43]. In our study, the average number of reported emotions (12.38 on average) is even slightly higher than the one observed by Sikka et al. [43] (7.24) using the same self-report scale. This discrepancy may be due to the fact that our scale includes two additional items or to a methodological difference. At variance with Sikka et al. [43], in our study participants were requested to complete the DREAM mDES immediately upon awakening, while dream reports were not requested. This methodological choice was made in order to assure that the recall process of dream emotions would be as unbiased as possible by the recall of dream content, and it possibly favored the detection of higher general emotionality in our participants’ dreams.

As for the predominant dream valence, Negative Affect (NA) scores were slightly higher than those at the Positive Affect (PA) subscale, but this difference failed to reach significance. The fact that general emotional tone of the dream appears relatively balanced is coherent with dream literature based on self-reports, which mostly shows a more balanced ratio of positive and negative emotionality (e.g., [42,60,69]) compared to studies using external coders (e.g., [41,70]). However, the analysis of discrete emotions showed that negative rather than positive emotions were both more frequent and more intense, in line with numerous studies that have addressed specific emotions (either self- [37,38,39] or externally rated [40,41]). Of note, this finding appears all the more significant when considering that our scale included twelve positive emotions, so that the risk of overestimating negative emotions is negligible.

More specifically, the fact that Fear appears among the most frequent and most intense emotions in our sample is in line with studies finding emotions in the fear domain (both self-reported and externally coded) to be the most frequently experienced in dreams (e.g., [37,38,40,41,51]). Along with Fear, Sadness and Awe also appear in our participants as frequently and intensely experienced emotions, again in accordance with existing results [41,42,43,51]. Similarly, our data show erotic feelings (Sensuality in our study) to be among the least frequently experienced dream emotions, as also previously observed in a few other studies [38,41].

### 4.3. Relationships between Wake and Dream Emotions

The main finding of our study is the profile of differences in positive and negative emotionality between waking emotions and dream emotions. While PA experienced the day before the dream was significantly reduced in the dream, NA displayed the inverse pattern. This profile, observed for general emotional valence of the dream, was also apparent from the analyses carried out on specific emotions, with most positive emotions reduced and most negative emotions increased in the dream relative to the previous day. Of note, although some comparisons did not reach significance, none of the emotions displayed changes in the opposite direction. Instead, as for emotions referred to the two preceding weeks, PA was significantly higher both relative to the day before the dream and to the dream itself, whereas NA was higher than that reported the day before the dream but did not differ from that of the dream. Again, the direction of data on specific emotions confirmed this trend, with most positive emotion scores decreasing from the two-weeks to the 24h time frame to the dream and most negative emotion scores higher than those of the day before the dream but displaying no differences with those of the dream. Interestingly, once more, none of the emotions showed changes in different directions.

Several observations may be made on these findings. First of all, when considering both the previous two-weeks and previous day time frames, positive emotionality is reduced in the dream, while negative emotionality is either increased (from the previous day) or stable (relative to the two weeks period). This suggests, as mentioned in the introduction, that valence differently affects the pathway of emotional expression from wake to the dream. A possible interpretation is that dream negative emotions reflect the negative emotions experienced more frequently during the general period in which the dream occurs (as indexed by equal intensity of NA and specific negative emotions in the dream and previous two weeks), expressing the sleep-related regulation mechanisms proposed by several theoretical models [14,15,16,17]. The observed increase of NA and specific negative emotions in the dream relative to the previous day is also compatible with these models [14,15,16,17]. In particular, in this case, we may refer to the recent hypothesis of a dream rebound of thoughts suppressed during wake [71,72,73], which, in turn, can be traced back to Freud’s idea [74] that dreams reflect the return of mental contents inhibited during the waking hours. In this perspective, it is plausible that negative emotions, consciously or unconsciously excluded from waking consciousness in favor of positive ones, rebound in the dream as an expression of their processing in the sleep state. In line with these hypotheses, positive emotionality would be underrepresented in the dream, compared to wake, since positive emotions require less regulation.

Another intriguing, complementary, hypothesis that comes to mind when looking at the general pattern of data is the existence of some sort of “day/night affective homeostasis” (an idea already suggested by [75]), with the dreaming experience of negative emotions balancing the prevalent expression of positive affect during wake time.

However, it must be noted that our regression analyses did not yield significant results. In fact, scores at the general affect scales (PA and NA) of the previous day and previous two weeks did not predict those of the dream; similarly, predictive relationships among corresponding wake and dream emotions emerged only for three emotions. These results are consistent with those of the three other studies assessing associations between dream and waking emotions [27,45,46]: Yu [45] found small to moderate correlations between corresponding dream and previous wake emotions; Gilchrist et al. [27] found that only a few waking emotions were strong predictors of dream emotions; Sikka et al. [46] did not find pre-sleep anger and interest to correlate with the corresponding emotions in the subsequent dream. Together with our results, this group of data do not suggest the existence of clear and direct relationships between waking and dream affect that could fully support the abovementioned hypotheses.

A couple of alternative explanations may be proposed to interpret our pattern of findings. One is that individuals could undergo some sort of social desirability effect in recognizing the experienced emotions from the scale (see, e.g., [76]); in other words, they would attribute to themselves, during wake, a majority of positive emotions (which are coherent with a positive image of the self), in a more or less deliberate manner [77]. Instead, negative emotions would be more easily attributed to a state of consciousness, such as the dream, which is experienced as involuntary and outside the boundaries of personal responsibility. This could explain the higher positive emotionality reported in both WAKE scales compared to negative emotionality and compared to the dream. A further hypothesis refers to a possible recall bias linked to the time frame of events to which the emotions refer. While in the WAKE-2wks mDES the participant is considering the past two weeks and in the WAKE-24hr version he is referring to the whole preceding day, in the DREAM mDES he is focusing on a much shorter time frame, that of the dream, which most probably includes a reduced number of events. Among this limited pool of memories, the negative ones could appear more salient and thus be more easily recognized (according to the widely held tenet in psychology that “bad is stronger than good”, i.e., more positive events are needed to overcome the salience of a single bad one, [78]). Moreover, it must be considered that our DREAM scale requires the subject to think back to the *last* recalled dream, which is probably an early morning REM sleep dream. This type of dreams have been suggested to be characterized by greater emotional negativity than earlier night dreams [41].

Another plausible hypothesis refers to the possibility that waking emotions are expressed in dreams across a different time span than those measured in our study. For instance, in analogy with data on the delayed incorporation of waking events in dreams [21,22,23], emotions experienced on a certain day could be reflected in the dream with a few days’ lag. However, our exploratory analysis on the participants reporting their dream with a few days’ delay from the beginning of data collection (and thus providing more than one WAKE24hr scale) does not support this idea. In fact, both PA and NA scores appeared relatively stable across the three days preceding the dream (i.e., similar to those of the day immediately preceding the dream). Due to the limited number of participants available for this analysis, correlations could not be assessed. Still, the possibility that waking emotions occurring at different time distances from the dream (e.g., several days, longer time periods than two weeks) may predict dream emotions should be investigated in future studies.

### 4.4. Limitations

Our results should be considered in light of some limitations to be overcome in future research. First of all, our choice of not collecting dream reports did not allow us to control for several dream features, such as dream length [41,79,80], which could have affected our results. Along the same lines, our participants’ ratings were collected at home, and we did not perform polysomnographic recordings to control for sleep stages and time spent in them, which also are believed to affect dream characteristics [81,82,83]. Furthermore, we cannot rule out that our results were affected by practice effects, linked to the repeated measurements performed using the same scale, i.e., participants’ ratings of one scale could have been modulated by their ratings of the previous one.

### 4.5. Conclusions

In conclusion, this study is the first to investigate the relationships of dream emotions with those experienced during the previous waking days using the same instrument, the modified Differential Emotions Scale, which assesses a broad array of positive and negative emotions and has proved to possess good psychometric properties in a preliminary validation study. Overall, our findings highlight a discontinuity between wake and dream affect, with positive emotionality reduced in the dream and negative emotionality similar to that of the preceding two weeks but increased relative to the previous day. In the frame of recent theoretical models postulating a role of dreaming in emotion regulation processes, these results suggest that positive and negative emotions experienced during wake may undertake different but parallel sleep-related regulation pathways. Although we did not observe strong direct relationships between wake and dream emotions, these findings also suggest the intriguing hypothesis of a “day/night affective homeostasis” to be addressed in forthcoming studies. Future research assessing wake and dream emotions over longer time periods and in different kinds of samples (healthy populations, individuals with emotional distress, clinical populations), as well as studies conducted with experimental rather than correlational designs, could be usefully implemented to further investigate this interesting possibility.

## Figures and Tables

**Table 1:** Mean ± SD of Positive and Negative Affect scores of the WAKE-2wks, WAKE-24hr, and Dream mDES.

	PA	NA	T	P_holm_
WAKE-2wks	1.97 ± 0.66	1.34 ± 0.51	3.91	0.001
WAKE-24hr	1.48 ± 0.78	0.82 ± 0.69	4.07	<0.001
Dream	0.96 ± 0.74	1.25 ± 0.80	−1.797	0.372

Notes: PA: Positive Affect; NA: Negative Affect.

**Table 2:** Statistics of the repeated measures ANOVA on each emotion.

Emotion	F_2,98_	*p*	*n_p_^2^*	WAKE-2wks vs. WAKE-24hr	WAKE-2wks vs. Dream	WAKE-24hr vs. Dream
*Amusement*	40.058	0.001	0.45	<0.001	<0.001	<0.001
*Anger*	13.52	0.014	0.22	<0.001	0.003	0.069
*Shame*	4.599	0.019	0.09	0.022	0.822	0.028
*Awe*	8.15	0.007	0.14	0.002	0.919	0.002
*Contempt*	3.806	0.035	0.07	0.042	0.803	0.053
*Disgust*	2.593	0.004	0.05	0.476	0.476	0.075
*Embarassment*	3.706	0.036	0.07	0.024	0.262	0.234
*Gratefulness*	29.23	0.004	0.37	0.001	<0.001	<0.001
*Guilt*	3.687	0.047	0.06	0.271	0.303	0.038
*Hatred*	6.33	0.065	0.06	0.316	0.345	0.059
*Hopefulness*	10.32	0.003	0.17	0.057	<0.001	0.021
*Inspiredness*	15.42	0.002	0.24	0.046	<0.001	0.002
*Interest*	18.92	0.002	0.27	0.13	<0.001	<0.001
*Joy*	12.48	0.007	0.2	0.098	<0.001	0.003
*Love*	15.5	0.003	0.24	0.096	<0.001	<0.001
*Pride*	19.62	0.004	0.29	<0.001	<0.001	0.011
*Sadness*	5.791	0.007	0.11	0.11	0.928	0.11
*Fear*	9.43	0.003	0.16	0.003	0.371	<0.001
*Serenity*	17.36	0.004	0.26	0.316	<0.001	<0.001
*Stress*	8.06	0.005	0.141	0.002	0.002	0.862
*Sensuality*	19.6	0.005	0.29	<0.001	<0.001	0.01
*Solidarity*	14.92	0.009	0.233	<0.001	<0.001	0.327

Notes: *p*-values of the ANOVA have been adjusted with the Benjamini–Hochberg approach. Holm test has been used for post-hoc comparisons and corrected *p*-values are reported.

## Supplementary Materials

The following are available online at https://www.mdpi.com/2076-3425/10/10/690/s1. S1. Validation study: Results. *S1.1. Item analysis. S1.2. Factor analysis. S1.3. Inter-item correlations. S1.4. Reliability analysis.* Table S1. Mean, Standard Deviation, Skewness, and Kurtosis of the Italian mDES items (both WAKE-24hr and WAKE-2wk forms). Table S2. Factor loading of the items of the Italian mDES, WAKE-24hr and WAKE-2wk forms. Table S3. Inter-item correlations of Positive Affect items of the Italian mDES (WAKE-24hr and WAKE-2wk forms). Table S4. Inter-item correlations of Negative Affect items of the Italian mDES (WAKE-24hr and WAKE-2wks forms). Table S5. Item-total correlations and Cronbach alpha values for Positive and Negative Affect items of the Italian mDES WAKE-24hr form. Table S6. Item-total correlations and Cronbach alpha values for Positive and Negative Affect items of the Italian mDES WAKE-2wk form. Table S7. Item-total correlations and Cronbach alpha values for Positive and Negative Affect items of the Italian mDES DREAM form.

## Appendix A

**Table A1:** Instructions and items of the DREAM mDES.

Modified Differential Emotions Scale (MDES)—Dream
Please think back to how you have felt during your last recalled dream. Using the 0–4 scale below, indicate the greatest amount that you’ve experienced each of the following feelings.
0 = Not at all, 1 = A little bit, 2 = moderately, 3 = Quite a bit, and 4 = Extremely
1. What is the most amused, fun-loving, or silly you felt?
2. What is the most angry, irritated, or annoyed you felt?
3. What is the most ashamed, humiliated, or disgraced you felt?
4. What is the most awe, wonder, or amazement you felt?
5. What is the most contemptuous, scornful, or disdainful you felt?
6. What is the most disgust, distaste, or revulsion you felt?
7. What is the most embarrassed, self-conscious, or blushing you felt?
8. What is the most grateful, appreciative, or thankful you felt?
9. What is the most guilty, repentant, or blameworthy you felt?
10. What is the most hate, distrust, or suspicion you felt?
11. What is the most hopeful, optimistic, or encouraged you felt?
12. What is the most inspired, uplifted, or elevated you felt?
13. What is the most interested, alert, or curious you felt?
14. What is the most joyful, glad, or happy you felt?
15. What is the most love, closeness, or trust you felt?
16. What is the most proud, confident, or self-assured you felt?
17. What is the most sad, downhearted, or unhappy you felt?
18. What is the most scared, fearful, or afraid you felt?
19. What is the most serene, content, or peaceful you felt?
20. What is the most stressed, nervous, or overwhelmed you felt?
21. What is the most sensual, excited, in mood for flirting you felt?
22. What is the most solidarity, care for the others, compassion you felt?
