# Supplementary material for: Relationships between Dream and Previous Wake Emotions Assessed through the Italian Modified Differential Emotions Scale
Source: Brain Sci. 2020 Sep 29;10(10):690. doi: 10.3390/brainsci10100690 (PMC7601812; doi:10.3390/brainsci10100690)
Supplement: Supplementary file 1 [file brainsci-10-00690-s001.pdf]

S1. Validation study: Results

S1.1. Item analysis

Table S1 shows mean, standard deviation, skewness and kurtosis for all 44 items making up the two forms of the scale (22 from the WAKE-24hr form, 22 from the WAKE-2wks form). Skewness and kurtosis ranged between -2 and +2 for all items, indicating normal distribution of the variables, except for item 3 (Shame) and 5 (Contempt) of the WAKE-24hr version (which may be due to these emotions being less intensely experienced or to a higher vulnerability to social desirability effects). In sum, item analysis showed no problematic items for either version of the Italian mDES.

Table S1. Mean, Standard Deviation, Skewness and Kurtosis of the Italian mDES items (both WAKE-24hr and WAKE-2wks forms).

Item	M	S.D.	Skewness	Kurtosis
WAKE-24hr form				
Item 1 (Amusement)	1.82	.988	-.07	-.56
Item 2 (Anger)	1.83	1.139	.07	-.87
Item 3 (Shame)	.50	.879	1.91	3.19
Item 4 (Awe)	1.14	1.095	.60	-.62
Item 5 (Contempt)	.62	.978	1.65	2.21
Item 6 (Disgust)	.71	1.011	1.56	1.93
Item 7 (Embarrassment)	.94	1.146	1.06	.18
Item 8 (Gratefulness)	1.95	1.118	-.09	-.63
Item 9 (Guilt)	.67	1.02	1.52	1.57
Item 10 (Hatred)	.90	1.121	1.07	.16
Item 11 (Hopefulness)	1.98	1.042	.02	-.45
Item 12 (Inspiredness)	1.71	1.15	.25	-.67
Item 13 (Interest)	2.38	.998	-.57	-.19
Item 14 (Joy)	1.93	1.026	.04	-.39
Item 15 (Love)	2.13	1.176	-.11	-.78
Item 16 (Pride)	1.90	1.043	.01	-.47
Item 17 (Sadness)	1.18	1.082	.79	-.07
Item 18 (Fear)	.97	1.118	.90	-.22
Item 19 (Serenity)	1.59	1.051	.18	-.52
Item 20 (Stress)	2.00	1.224	.11	-.97
Item 21 (Sensuality)	1.27	1.247	.64	-.63
Item 22 (Solidarity)	1.97	1.135	-.20	-.75

WAKE-2wks form				
Item 1 (Amusement)	2.29	.864	-.24	-.20
Item 2 (Anger)	2.32	.881	.09	-.55
Item 3 (Shame)	.96	.978	1.03	.68
Item 4 (Awe)	1.76	.980	.04	-.53
Item 5 (Contempt)	.84	.988	1.18	1.04
Item 6 (Disgust)	.99	1.053	.76	-.47
Item 7 (Embarrassment)	1.38	1.030	.29	-.73
Item 8 (Gratefulness)	2.23	.895	-.50	.35
Item 9 (Guilt)	.93	1.064	1.11	.63
Item 10 (Hatred)	1.24	1.140	.53	-.79
Item 11 (Hopefulness)	2.03	.943	-.09	-.33
Item 12 (Inspiredness)	1.97	.985	-.02	-.56
Item 13 (Interest)	2.45	.935	-.45	-.21
Item 14 (Joy)	2.32	.938	-.29	-.17
Item 15 (Love)	2.47	1.060	-.54	-.26
Item 16 (Pride)	2.11	.984	-.07	-.33
Item 17 (Sadness)	1.85	.937	.06	-.37
Item 18 (Fear)	1.43	1.035	.17	-.82
Item 19 (Serenity)	1.97	.971	.04	-.57
Item 20 (Stress)	2.42	.933	-.17	-.47
Item 21 (Sensuality)	1.85	1.125	-.16	-.85
Item 22 (Solidarity)	2.31	.962	-.34	-.12

Notes. N = 212. Answer Range = 0 to 4.

S1.2. Factor analysis

Based on previous literature [55,58,59], a bifactorial structure was assumed for both mDES versions, reflecting the positive or negative emotional valence of the items. First, in analogy with Galanakis et al. [55], a Principal Components Analysis with orthogonal rotation (normalized Varimax) was conducted for each of the two forms of the scale (WAKE-24hr and WAKE-2wks). Results confirmed the bifactorial structure for both forms (Factor 1: Positive Affect, Factor 2: Negative Affect), with an explained variance of 42.59% for the WAKE-24hr version and of 41.59% for the WAKE-2wks version (Table S2).

A robust confirmatory factor analysis was carried out to test the bi-dimensionality of the 22-item version of both scales. As fit indices, we used the maximum likelihood chi-square test (MLχ2), the root mean square error of approximation index (RMSEA); and the comparative fit index (CFI). Results confirmed that the 2-factor model had adequate fit indices for both versions of the scale: WAKE-24hr, RMSEA = .066 90%CI

[.06; .08]; CFI = .955, MLc2(207, N = 200) = 702.64, p < .001, and WAKE-2wks, RMSEA = .069 90%CI [.06; .08]; CFI = .950, MLc2(205, N = 200) = 700.17, p < .001.

Table S2. Factor loading of the items of the Italian mDES, WAKE-24hr and WAKE-2wks forms.

**Item**	**WAKE-24hr**			**WAKE-2wks**	
	Factor 1 (Positive Affect)	*Factor 2 (Negative Affect)*		*Factor 1 (Positive Affect)*	*Factor 2 (Negative Affect)*
Item 1 (Amusement)	.561	-.219		.601	-.156
Item 2 (Anger)	-.161	.688		-.068	.647
Item 3 (Shame)	.127	.536		-.023	.503
Item 4 (Awe)	.515	.150		.534	.140
Item 5 (Contempt)	-.072	.512		.047	.507
Item 6 (Disgust)	-.073	.674		-.104	.671
Item 7 (Embarrassment)	.340	.472		.165	.386
Item 8 (Gratefulness)	.659	.091		.649	.108
Item 9 (Guilt)	.037	.587		-.056	.677
Item 10 (Hatred)	-.107	.717		-.065	.664
Item 11 (Hopefulness)	.760	-.105		.736	-.087
Item 12 (Inspiredness)	.657	-.001		.649	-.182
Item 13 (Interest)	.626	-.210		.684	-.203
Item 14 (Joy)	.786	-.278		.775	-.180
Item 15 (Love)	.657	-.205		.684	-.092
Item 16 (Pride)	.527	-.114		.625	-.148
Item 17 (Sadness)	-.255	.749		-.315	.719
Item 18 (Fear)	-.026	.706		-.273	.587
Item 19 (Serenity)	.654	-.402		.679	-.270
Item 20 (Stress)	-.168	.650		-.133	.623
Item 21 (Sensuality)	.463	.080		.572	-.014
Item 22 (Solidarity)	.470	.125		.414	.195
Proportion of explained variance	*.222*	*.204*		*.235*	*.181*

Notes. N = 212.

S1.3. Inter-item correlations

Pearson's correlations among the items saturating on the two factors (Positive Affect and Negative Affect) in both the WAKE-24hr and WAKE-2wks mDES forms confirmed the good construct validity of the

scale, with average inter-item correlation values all between .20 and .40 [58] for both forms of the scale. Specifically, for the WAKE-24hr version, average inter-item correlations for Factor 1 (Positive Affect) and Factor 2 (Negative Affect) were .339 and .343, respectively, and those for the WAKE-2wks version were .362 and .307, respectively.

In both mDES versions, almost all correlations between items loading on both factors (Positive Affect and Negative Affect) were statistically significant ($p<.05$). Correlation values for positive emotion items (both scale forms) and negative emotion items (both scale forms) are displayed in Tables S3 and S4, respectively.

Table S3. Inter-item correlations of Positive Affect items of the Italian mDES (WAKE-24hr and WAKE-2wks forms).

ITEM	1	4	8	11	12	13	14	15	16	19	21	22
**1**		.207**	.250***	.415***	.304***	.293***	.549***	.347***	.211**	.384***	.322***	.135*
**4**	.334***		.319***	.281***	.228**	.256***	.388***	.229**	.095	.197**	.163*	.259***
**8**	.301***	.311***		.528***	.300***	.335***	.419***	.412***	.199**	.427***	.156*	.354***
**11**	.380***	.402***	.391***		.514***	.501***	.562**	.424***	.382***	.472***	.242***	.268***
**12**	.323***	.252***	.320***	.536***		.516***	.421***	.274***	.351***	.388***	.308***	.176*
**13**	.315***	.253***	.320***	.539***	.618***		.448***	.362***	.393***	.415***	.171*	.219**
**14**	.501***	.336***	.462***	.514***	.406***	.513***		.589***	.409***	.659***	.289***	.251***
**15**	.355***	.246***	.408***	.428***	.356***	.391***	.604***		.320***	.499***	.312***	.308***
**16**	.314***	.297***	.284***	.446***	.395***	.426***	.491***	.374***		.424***	.292***	.258***
**19**	.424***	.246***	.456***	.482***	.351***	.450***	.615***	.462***	.376***		.233**	.173*
**21**	.362***	.186**	.255***	.299***	.359***	.348***	.355***	.413***	.306***	.360***		.150*
**22**	.160*	.150*	.385***	.199**	.206**	.224**	.163*	.238***	.204**	.143*	.162*	

Notes. Items of the WAKE-24hr form appear above the diagonal, whereas those of the WAKE-2wks form are shown below the diagonal. N = 212.
$^{*}p<.05$; $^{**}p<.01$; $^{***}p<.001$

Table S4. Inter-item correlations of Negative Affect items of the Italian mDES (WAKE-24hr and WAKE-2wks forms).

ITEM	2	3	5	6	7	9	10	17	18	20
**2**		.256***	.376***	.504***	.169*	.290***	.495***	.490***	.335***	.513***

**3**	.295***		.107	.204**	.480***	.368***	.248***	.288***	.333***	.216**
**5**	.209**	.195**		.391***	.020	.223**	.346***	.391***	.255***	.325***
**6**	.371***	.210***	.504***		.151*	.279***	.560***	.420***	.454***	.364***
**7**	.066	.400***	-.017	.108		.269***	.172***	.170*	.298***	.210**
**9**	.285***	.275***	.292***	.262***	.282***		.365***	.391***	.373***	.224**
**10**	.506***	.182**	.336***	.507***	.097	.353***		.531***	.418***	.387***
**17**	.403***	.247***	.282***	.445***	.212**	.527***	.406***		.529***	.526***
**18**	.218**	.218**	.164*	.330***	.219**	.482***	.242***	.537***		.426***
**20**	.516***	.148*	.123	.313***	.099	.343***	.409***	.469***	.373***	

Notes. Items of the WAKE-24hr form appear above the diagonal, whereas those of the WAKE-2wks form are shown below the diagonal. N = 212.
$^{*}p$<.05; $^{**}p$<.01; $^{***}p$<.001

S1.4. Reliability analysis

The reliability analysis (item-total correlations) conducted on the items loading on the two factors (Positive Affect and Negative Affect) for both forms of the scale (WAKE-24hr and WAKE-2wks) sustained a good internal consistency of the instrument, with all Cronbach alpha values >.77 (Tables S5 and S6). Table S7 also shows results of the same analysis conducted on the items of the DREAM mDES, collected from the 50 participants of the second part of the study (dream study): all Cronbach alpha values were >.80.

Table S5. Item-total correlations and Cronbach alpha values for Positive and Negative Affect items of the Italian mDES WAKE-24hr form.

WAKE-24hr mDES - Positive Affect items

Cronbach Alpha = .857
Average inter-item correlation = .339

**ITEM**	**Item-Tot Correlation**	**Alpha if Delete**
Item 1 (amused, fun-loving, or silly	.493	.844
Item 4 (awe, wonder, or amazement)	.373	.852
Item 8 (grateful, appreciative, or thankful)	.536	.841
Item 11 (hopeful, optimistic, or encouraged)	.676	.832
Item 12 (inspired, uplifted, or elevated)	.545	.841
Item 13 (interested, alert, or curious)	.568	.839
Item 14 (joyful, glad, or happy)	.740	.828

Item 15 (love, closeness, or trust)	.600	.837
Item 16 (proud, confident, or self-assured)	.480	.845
Item 19 (serene, content, or peaceful you)	.623	.835
Item 21 (sensual, excited, in mood for flirting)	.375	.854
Item 22 (solidarity, care for the others, compassion)	.363	.854

WAKE-24hr mDES - Negative Affect items
Cronbach Alpha = .835
Average inter-item correlation = .343

Item 2 (angry, irritated, or annoyed)	.605	.813
Item 3 (ashamed, humiliated, or disgraced)	.430	.829
Item 5 (contemptuous, scornful, or disdainful)	.418	.830
Item 6 (disgust, distaste, or revulsion)	.586	.815
Item 7 (embarrassed, self-conscious, or blushing)	.318	.842
Item 9 (guilty, repentant, or blameworthy)	.474	.825
Item 10 (hate, distrust, or suspicion)	.620	.811
Item 17 (sad, downhearted, or unhappy)	.667	.810
Item 18 (scared, fearful, or afraid)	.601	.813
Item 20 (stressed, nervous, or overwhelmed)	.561	.817

Notes. N = 212.

Table S6. Item-total correlations and Cronbach alpha values for Positive and Negative Affect items of the Italian mDES WAKE-2wks form.

WAKE-2wks mDES - Positive Affect items

Cronbach Alpha = .869
Average inter-item correlation = .362

ITEM	Item-Tot Correlation	Alpha if Delete
Item 1 (amused, fun-loving, or silly	.532	.858
Item 4 (awe, wonder, or amazement)	.414	.866
Item 8 (grateful, appreciative, or thankful)	.549	.857
Item 11 (hopeful, optimistic, or encouraged)	.660	.850
Item 12 (inspired, uplifted, or elevated)	.584	.855
Item 13 (interested, alert, or curious)	.628	.852
Item 14 (joyful, glad, or happy)	.715	.847
Item 15 (love, closeness, or trust)	.609	.853

Item 16 (proud, confident, or self-assured)	.553	.857
Item 19 (serene, content, or peaceful you)	.620	.852
Item 21 (sensual, excited, in mood for flirting)	.477	.863
Item 22 (solidarity, care for the others, compassion)	.304	.872

WAKE-2wks mDES - Negative Affect items
Cronbach Alpha = .811
Average inter-item correlation = .307

Item 2 (angry, irritated, or annoyed)	.520	.790
Item 3 (ashamed, humiliated, or disgraced)	.400	.802
Item 5 (contemptuous, scornful, or disdainful)	.376	.805
Item 6 (disgust, distaste, or revulsion)	.574	.783
Item 7 (embarrassed, self-conscious, or blushing)	.256	.818
Item 9 (guilty, repentant, or blameworthy)	.567	.784
Item 10 (hate, distrust, or suspicion)	.550	.786
Item 17 (sad, downhearted, or unhappy)	.656	.776
Item 18 (scared, fearful, or afraid)	.503	.791
Item 20 (stressed, nervous, or overwhelmed)	.502	.792

Notes. N = 212.

Table S7. Item-total correlations and Cronbach alpha values for Positive and Negative Affect items of the Italian mDES DREAM form.

DREAM mDES - Positive Affect items
M = 11.56; *SD* = 8.83
Cronbach Alpha = .896
Average inter-item correlation = .435

ITEM	Item-Tot Correlation	Alpha if Delete
Item 1 (amused, fun-loving, or silly)	.623	.884
Item 4 (awe, wonder, or amazement)	.189	.906
Item 8 (grateful, appreciative, or thankful)	.617	.884
Item 11 (hopeful, optimistic, or encouraged)	.697	.879
Item 12 (inspired, uplifted, or elevated)	.764	.876
Item 13 (interested, alert, or curious)	.521	.889
Item 14 (joyful, glad, or happy)	.743	.876
Item 15 (love, closeness, or trust)	.757	.875

Item 16 (proud, confident, or self-assured)	.724	.878
Item 19 (serene, content, or peaceful you)	.791	.875
Item 21 (sensual, excited, in mood for flirting)	.486	.890
Item 22 (solidarity, care for the others, compassion)	.420	.896

DREAM mDES - Negative Affect items
M = 12.52; *SD* = 8.03
Cronbach Alpha = .839
Average inter-item correlation = .349

Item 2 (angry, irritated, or annoyed)	.599	.817
Item 3 (ashamed, humiliated, or disgraced)	.616	.817
Item 5 (contemptuous, scornful, or disdainful)	.501	.828
Item 6 (disgust, distaste, or revulsion)	.688	.808
Item 7 (embarrassed, self-conscious, or blushing)	.249	.849
Item 9 (guilty, repentant, or blameworthy)	.186	.851
Item 10 (hate, distrust, or suspicion)	.675	.809
Item 17 (sad, downhearted, or unhappy)	.739	.802
Item 18 (scared, fearful, or afraid)	.484	.829
Item 20 (stressed, nervous, or overwhelmed)	.563	.821

Notes. N = 50.
